# Relationship between Stress Shielding and Optimal Femoral Canal Contact Regions for Short, Tapered-Wedge Stem Analyzed by 2D and 3D Systems in Total Hip Arthroplasty

**DOI:** 10.3390/jcm12093138

**Published:** 2023-04-26

**Authors:** Takashi Maeda, Masaki Nakano, Yukio Nakamura, Takashige Momose, Atsushi Sobajima, Jun Takahashi, Katsuya Nakata, Masashi Nawata

**Affiliations:** 1 Department of Orthopaedic Surgery, Marunouchi Hospital, Matsumoto 390-8601, Japan; 2 Department of Orthopaedic Surgery, Shinshu University School of Medicine, 3-1-1 Asahi, Matsumoto 390-8621, Japan; 3 Department of Orthopaedic Surgery, JCHO Osaka Hospital, Fukushima-ku, Osaka 553-0003, Japan

**Keywords:** anterolateral supine approach, stress shielding, tapered-wedge short stem, three-dimensional analysis, total hip arthroplasty

## Abstract

Although tapered-wedge short stem has been widely employed with its availability for minimally invasive surgeries in total hip arthroplasty (THA), post-operative stress shielding matter remains unresolved in cementless procedures. This study aimed to clarify the most optimal femoral canal contact regions of the stem design taking stress shielding incidence into consideration. This investigation included 60 joints from 60 patients (mean age at operation: 65.9 years), of which follow-up duration after primary THA had been more than 2 years. Frequencies of spot welds, subsidence, and stress shielding were examined 2 years after surgery. The most suitable femoral canal contact regions were evaluated by plain radiograph (2D) and 3D-computed tomography analyses according to Nakata’s division for fitting manners. Spot welds were observed in 38 cases (63.3%), and no subsidence case was seen. Respective number of stress shielding cases, based on Engh’s classification, categorized as degree 0, 1, and 2, were 2 (3.3%), 31 (51.7%), and 27 (45.0%), while no cases for degree 3 or 4 were found. When assessed by 3D fitting analysis, 27 cases of stress shielding degree 2 were constituted by 13/42 cases of mediolateral (ML) fit, 2/4 cases of flare fit, and 12/14 cases of multi point fit. In 42 cases of ML fitting, stem contact rate of the most proximedial region in stress shielding degree 0 and 1 was significantly higher compared to stress shielding degree 2 cases. Meanwhile, the rates of distal regions were significantly lower or absent in stress shielding degree 0 and 1 cases. The initial fixation of this stem design was very good in our cohort regardless of fitting manners. This study successfully revealed that ML fitting with femoral component, especially the most proximedial calcar site restricted fitting, would be optimal for reducing stress shielding occurrence in cementless short, tapered-wedge stem THA. Thus, the ideal stem contact region should be considered during THA procedures in light of the reduction of stress shielding development.

## 1. Introduction

Restoring the condition of the inflamed hip joint through surgery with total hip replacement is a surgical intervention that is highly effective today. Recently, the number of total hip arthroplasty (THA) cases employing cementless fixation of acetabular and femoral components has been increasing worldwide, while the femoral stem designs are differing in material, surface coating, and shape. Material coupling on the sliding surface reportedly affects Tresca stress under gait cycles [1]. Implant shape is a key factor for defining the regions of cortical contact, where the initial fixation will be obtained. Among a variety of femoral stem types including tapered-wedge stem, short type of cementless stem has been developed with the advantages of insertion through smaller skin incision as well as preservation of metaphyseal and diaphyseal bone stocks and possibly ensuing a decrease in thigh pain [2]. However, stress shielding after THA procedures has drawn much attention in clinical settings. Improvements in cementless stem fixation could have resulted in raising stress shielding matters in the proximal femur.

There have been some previous reports that the occurrence and degrees of stress shielding were different from those based on the proximal fitting or metaphyseal to distal cementless stem designs [3,4,5,6,7]. As for the proximal filling stems, the occurrence of stress shielding using Synergy stem (Smith & Nephew, Watford, UK) or modular femoral sleeve and stem (S-ROM; DePuy Orthopaedics, Warsaw, IN, USA) was 84% or 42%, respectively [3,4]. On the other hand, stress shielding incidence caused by metaphyseal-fitting anatomic stem, CLS femoral prosthesis (Zimmer Biomet, Warsaw, IN, USA), and Taperloc femoral component (Zimmer Biomet, Warsaw, IN, USA), those of which were metaphyseal to distal fitting stems, were 6%, 0%, and 3%, respectively [5,6,7]. These reports suggest that the occurrence of stress shielding may differ among materials, length, or fixation concept of each stem. As little occurrence as possible of stress shielding in THA procedure has been a great challenge for joint surgeons over many years.

Short and tapered-wedge stem has been believed to provide lesser stress shielding development due to its geometry. However, there exist some cases with strong stress shielding after THA, though using this stem design, in clinical settings. Few studies have focused on the investigation of relationship between stem contact regions and stress shielding occurrence after cementless THA procedure until now [8]. In general, the concept for obtaining initial stability of tapered-wedge stem is wedge fixation at flare regions and three-point fixation [9]; however, it has not been elucidated whether the initial fixation concept correlates with adequate bone reaction or not. To avoid revision surgeries owing to periprosthetic fracture, optimal stem contact regions for reducing stress shielding development should be investigated. In this regard, we hypothesized that there exists a relationship between the manner of initial fixation and bone response. Herein, we report the first kind of study to clarify the association of the most optimal contact regions of short, tapered-wedge stem analyzed by two- or three-dimensional imaging with the incidence of stress shielding after cementless THA.

## 2. Methods

This study was designed to clarify the most suitable fitting region of femoral stem for reducing stress shielding as much as possible in cementless tapered-wedge short stem THA. The present investigation retrospectively included all patients who had undergone primary THA performed by multiple certified orthopedic surgeons with anterolateral (AL) supine approach at Marunouchi Hospital, Japan, between June 2016 and July 2017. Sixty-two joints from 62 patients were enrolled originally; however, 2 cases were lost to follow-up due to non-visit to our facilities. There were no patients excluded from this investigation. Finally, 60 joints from 60 patients (10 male and 50 female), of which follow-up duration after surgery had been more than 2 years, were analyzed. All subjects were diagnosed with primary hip osteoarthritis, and the average follow-up period was 31.8 ± 3.2 months. In all cases, a short, tapered-wedge stem (Taperloc Microplasty; Zimmer Biomet, Warsaw, IN, USA) and the Continuum Acetabular System (Continuum Multi-Hole Shell, Neutral Liner, Biolox Delta Ceramic Head; Zimmer Biomet, Warsaw, IN, USA) were used for THA.

**Figure 1:**
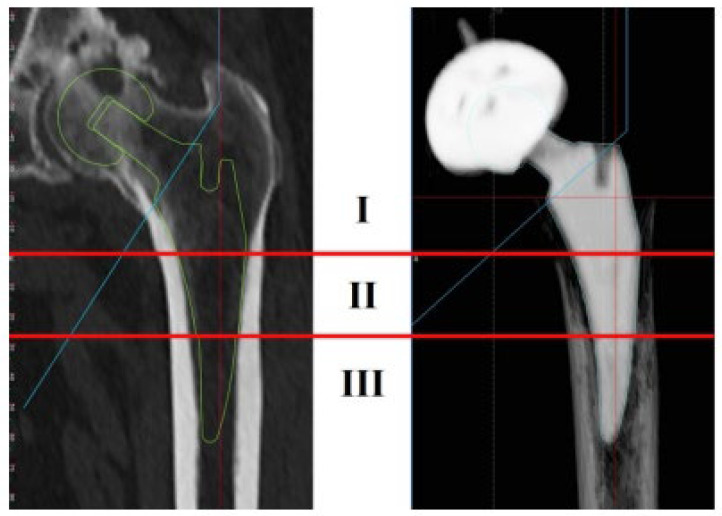
Diagram of stress shielding degree classification. 1, rounding-off of the proximedial femoral neck; 2 and 3, loss of medial cortex density at level I and II (around and below the lesser trochanter); 4, cortical resorption into the diaphysis.

**Figure 2:**
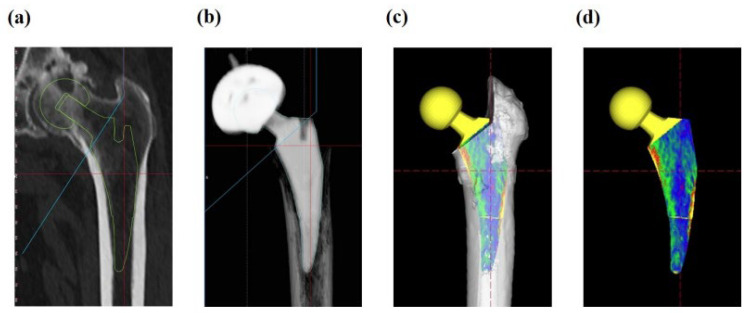
Fitting assessment of femoral component. Three-dimensional computed tomography (3D-CT) image (**a**) before and (**b**) 3 months after surgery. (**c**,**d**), Computer-aided diagnosis model of the placed stem reproduced in pre-surgery 3D-CT image based on the after-surgery information.

Based on pre-surgery plain radiographs, each femoral canal morphology was assessed by canal flare index (CFI) [10], while spot welds [11], subsidence, and stress shielding were examined by plain radiographs after 2 years of surgery. We evaluated the stress shielding degree by using Engh’s classification (1–4 degree rating) [12], and the case with no stress shielding signs was defined as stress shielding degree 0 (Figure 1). Radiographic imaging was conducted by KXO-50S (Toshiba Medical Systems, Tochigi, Japan). Three-dimensional computed tomography (3D-CT) was carried out before surgeries and at 3 months after surgeries, and the image data were analyzed by ZedHip software (Lexi, Tokyo, Japan). ZedHip is a 3D preoperative planning software using CT imaging for THA procedure, and it enables us to select the size of stem/acetabular cup and examine 3D placement position within 15 min, which is radial measurement time, by employing a unique range of motion simulation system. CT imaging was performed by Aquilion ONE (Canon Medical Systems, Tochigi, Japan) with 320-row area detector, and CT dose index was 7 millisievert (mSv). We had conducted pre- and post-surgery CT scan and plain radiograph. Position information of each inserted stem was extracted from 3D-CT computer-aided diagnosis (CAD) model (Lexi, Tokyo, Japan) after surgery; then, the placed stem position was reproduced in pre-surgery 3D-CT based on the after-surgery information. Accuracy of the 3D-CT modeling has been characterized in the literature [13]. The border between femoral cortical and cancellous bones was defined as 600 Hounsfield units (HU) of 3D-CT value. Since the definition of 600 HU for cortical bone is very common biomechanically, the regions of CAD model surface displaying 600 HU or more were determined as contact regions of each stem with femoral bone (Figure 2). Workflow of the present study is summarized in Supplemental Figure S1.

**Figure 3:**
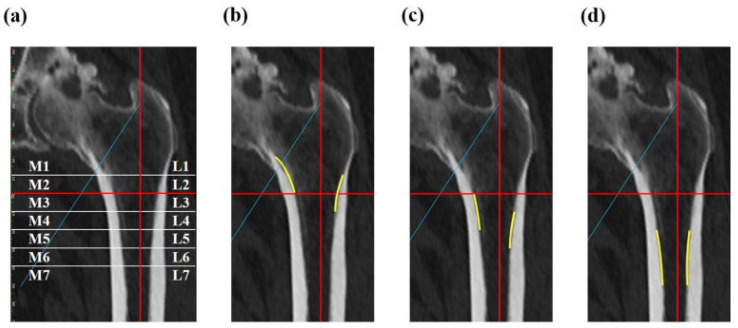
Definition of stem contact region on femoral canal. (**a**) According to Nakata’s division [14], seven regions of both inner and outer sides were defined (M1–M7 and L1–L7). (**b**) Mediolateral fit. (**c**) Flare fit. (**d**) Diaphyseal fit.

Contact regions between stem and femoral canal were assessed and classified according to Nakata’s division [14]. We defined the fit condition, such as minimal level of contact, based on pre-operative 3D-CT image and CAD model. Briefly, 7 regions of both inner (from the proximal medial site of femur, M1 to M7) and outer sides (from the proximal lateral site of femur, L1 to L7) were defined from the center of lesser trochanter to proximal (zones 1–2) or distal sites (zones 3–7) by every 10 mm [14]. The most suitable femoral canal regions were evaluated by plain radiograph (2D) and 3D-CT analyses. Moreover, the occurrence of stress shielding according to the stem fit conditions was examined. The manner of stem fitting was defined as mediolateral fit (ML, M1–M2 and L2–L3), flare fit (FLR, M3–M4 and L4–L5), diaphyseal fit (M5–M7 and L5–L7), and multi point fit (MULTI) (Figure 3). Stress shielding degrees of 0, 1, and 2 were observed in the cohort. Statistical analysis was performed by using the chi-squared test. A two-tailed *p*-value of < 0.05 was considered significantly different.

In addition to the information disclosure for opt-out on the website, comprehensive informed consent of each patient for the research and publication was obtained verbally, and its statement was written in the medical reports at registration, and minors were not involved in this study. The present study was approved by the Institutional Review Board of Marunouchi Hospital, Japan (approval number: 21-4). The research procedure was conducted in accordance with the ethical guidelines of the 2013 Declaration of Helsinki.

## 3. Results

**Figure 4:**
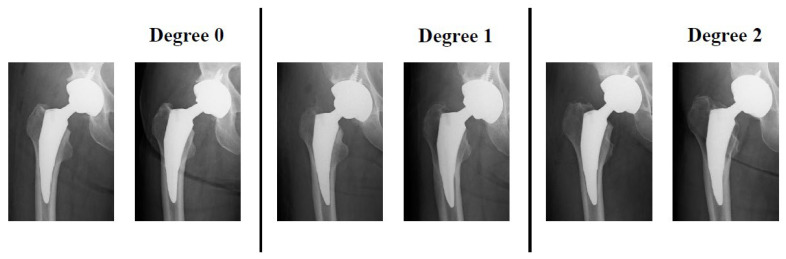
Representative radiographic image for each stress shielding degree. Left panel, immediately after surgery; Right panel, 2 years after surgery.

The mean ± standard deviation (SD) age in all of the patients at operation was 65.9 ± 11.5 years (31–87 y/o). The average ± SD score of CFI was 3.56 ± 0.7 (normal, forty-six cases (76.7%); champagne-flute, eleven cases (18.3%); stovepipe, three cases (5.0%)). The development of spot welds was observed in 38 cases (63.3%), in which zones 2, 3, 5, or 6 corresponded to 15 cases (25.0%), 25 cases (41.7%), 16 cases (26.7%), or 20 cases (33.3%), respectively. The zones were defined on the basis of Nakata’s division (see Figure 3a) [14]. No subsidence was seen in all cases. Regarding the occurrence of stress shielding, the respective number of cases categorized as degree 0, 1, and 2 were 2 (3.3%), 31 (51.7%), and 27 (45.0%), while no cases for degree 3 or 4 were found among our 60 cases. Representative radiographic image for each stress shielding degree is shown in Figure 4.

The manner of stem fitting according to 2D-radiographic analysis demonstrated forty-six, nine, and five cases in ML, FLR, and MULTI, respectively, whereas 3D-CT analysis showed forty-two, four, and fourteen cases in corresponding manners. In these analyses, the difference in contact region assessment between 2D and 3D procedure was observed in 13 cases. These assessment changes from 2D to 3D analysis included eight cases from ML to MULTI, one case from FLR to MULTI, and four cases from FLR to ML. Good initial fixation was obtained in all manners of stem fitting; however, bone response differed among them. When assessed by 3D fitting analysis, 27 cases of stress shielding degree 2 were constituted by 13/42 cases of ML, 2/4 cases of FLR, and 12/14 cases of MULTI.

**Figure 5:**
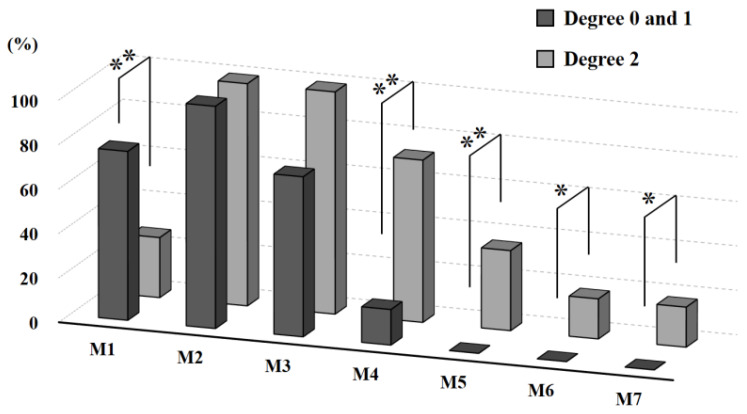
Stem contact regions according to stress shielding degree. Contact regions of each stem among medial sites of femur were examined using three-dimensional system. Significance of differences was evaluated by chi-squared testing (* *p* < 0.05 and ** *p* < 0.01).

In 42 cases of ML fitting evaluated by 3D-CT, CFI of stress shielding degree 0 and 1 (3.47 ± 0.6) was not significantly different compared to stress shielding degree 2 cases (3.43 ± 0.7). Regarding these ML fitting cases, examination for contact regions of each stem among medial sites of femur, M1 to M7, revealed that the rate of M1 region in stress shielding degree 0 and 1 was significantly higher than that in stress shielding degree 2 cases. On the other hand, the rates of M4, 5, 6, and 7 regions were significantly lower or absent in stress shielding degree 0 and 1 cases (Figure 5). Moreover, when the contact regions were examined according to two, three, and four or more sites, we found a significantly higher incidence rate of stress shielding degree 2 in the cases of four or more contact sites (Table 1). Two in sixty cases (3.3%) showed no stress shielding based on the area of fit.

## 4. Discussion

This study is the first to reveal the most ideal fitting region during cementless THA with tapered-wedge short stem for reducing stress shielding as much as possible, and thereby our findings may lead to the improvement of THA procedure and development of newly-designed stem. Our findings are the first kind of study using tapered-wedge short stem in light of clarification of the most suitable fitting region of femoral stem, which have revealed that ML fit of the stem would be optimal in THA. Other researchers have reported on similar results in which stress shielding frequencies less likely occur by ML fit during THA procedure using calcar loading short stem, based on plain radiograph and/or bone mineral density [15,16,17].

The stability and durability of femoral stems after cementless THA procedure are influenced by the location of inserted stem in femur. THA with short stem contributes to the preservation of femoral bone stock and is believed to provide a more natural loading compared with standard stem designs [18,19]. Therefore, short-stem THA has primarily been recommended for relatively young or active patients and some elderly patients as well. However, because of a variety of femoral canal morphology, the optimal fitting between prosthesis and femur is not necessarily obtained. In the present study, we determined the most appropriate femur-stem placement site by 3D analysis, which takes into account the development of stress shielding in short, tapered-wedge cementless stem design. Our data with stress shielding evaluation clarified that the optimal short stem fitting location in femur would be M1, or at least M1–M3 regions, while fitting with a wide range of regions might be better avoided. Note that how contact region is defined will be important since excessive decrease in contact regions may diminish initial fixation of implants, although it is important to decrease contact regions due to the avoidance of stress shielding occurrence.

Stress shielding is also related to Young’s modulus of elasticity of the stem. It is considered that a short, tapered-wedge stem (Taperloc Microplasty; Zimmer Biomet, Warsaw, IN, USA) is composed of titanium alloy and small rigidity, expecting that the occurrence of stress shielding might be few compared with that caused by cobalt-chrome (CoCr)-containing THA regardless of its design. Our findings in this study have suggested that the occurrence of stress shielding would depend on the fitting regions. Engh and colleagues previously reported on the biological fixation of porous-surfaced femoral components, one-third porous-coated anatomic medullary locking (AML) prosthesis, evaluated by plain radiographs. They have described that (1) The degrees of stress shielding depend on the occurrence sites of spot welds; (2) The occurrence of spot welds seems to be a sign of bone ingrowth or ongrowth; (3) Proximal or distal fixation can be determined based on the sites of spot welds; (4) The load from femoral component leads to further distal cortical bone via spot welds, and then stress shielding occurs at more proximal sites than the site of spot welds [20]. Therefore, it is likely considered that stress shielding is a kind of bone reaction caused by spot welds, which is a result of good implant fixation. Additionally, stress shielding could be a cause of periprosthetic fracture (PPF) [21] or increases the bone injuries and fractures at revision THA instead of femoral component loosening.

Toward gaining better survival rates of THA, the most optimal contact region with femoral component should be defined since this concept has never been advocated previously. Stress shielding matter remains unresolved in most THA cases, even with newer stem designs; thus, we addressed the issue in light of stress shielding occurrence by using 2D and 3D systems. It is considered that stress shielding could be related to PPF since poor bone stock caused by stress shielding is a risk factor for PPF occurrence [21]. So far, nearly half of cases with the development of stress shielding degree 2 or more have been reported as 50.5% by Engh et al. [12] and 62.5% by Tetsunaga et al. [22], and those were slightly higher compared with our observation (45.0%), suggesting that the occurrence of stress shielding might differ among materials, length, or concept of fixation of each stem. As for the femoral canal morphology of patients, when compared with the report by Noble et al. [10], our cohort had less normal type (77% vs. 83%) but more champagne-flute type (18% vs. 8%) or less stovepipe type (5% vs. 9%). In the current study, no stem subsidence and more than half of cases with spot welds (63.3%) were observed, thereby suggesting that the initial fixation was good. However, there existed stress shielding degree 2 cases in nearly half of our cohort. Stress shielding often occurs in the calcar region of femur and causes poor bone density and quality [23]. Consequently, stress shielding may be a major cause of PPF after THA procedure. Therefore, implant fixation should aim to reproduce physiological stress distribution.

The concept for initial fixation of tapered-wedge stem has been proximal metaphyseal fitting [9]. Very recently, Li et al. proposed that the AML prosthesis (DePuy Orthopaedics, Warsaw, IN, USA) would bring a good bone remodeling and long-term effectiveness with a median of 16.5 years’ follow-up, suggesting better long-term results with appropriate bone fitting in proximal medial region [24]. The Zimmer Taperloc stem has reduced geometry, which enhances the proximal fitting of the implant in the femoral metaphysis; its shape and design may pertain to our data. In our cohort, good initial fixations were equally obtained in ML, FLR, and MULTI fitting manners. However, our findings showed a tendency that the more fitting sites became widely available, the stress shielding of degree 2 or more likely occurred on the sites from M1 to M4. In addition, as shown in Table 1, the more the number of fitting areas increased, the stress shielding degree became worse. These findings suggest that wide sites fitting of femoral stem implant may cause the increased number of distal spot welds, and thereby lead to a higher degree of stress shielding. Thus, this study with short and tapered-wedge stem demonstrated that ML fitting could be considered as the optimal fitting. In addition, our study suggested that the development and progression of stress shielding could be avoided if the stem fitted M1 region among ML fitting manner. These findings imply that the most ideal stem contact region will be at M1, or at least restricted to M1–M3 regions. Sas et al. reported that stress shielding was found to be the highest in the proximal regions on the medial and posterior sides of the calcar loading short stem, based on computational simulation/in silico, which means that ML fit of the stem can reduce bone atrophy [18]. However, they have used calcar loading short stem, not our tapered-wedge short stem. In any cases, it is conceivable that ML fit of femoral stem will be optimal in terms of bone stock during THA. Note that there may be respective optimal contact sites according to the femoral canal morphology. Therefore, future studies are needed on this notion.

The present study included some strengths and several limitations. The strength of this study was the identification of the most optimal fitting region of short, tapered-wedge stem based on the occurrence of stress shielding during cementless THA. On the other hand, the limitations of the current study were as follows: First, this investigation was a single-centered retrospective study that might have overlooked some information and potential risk factors. Second, this study had a relatively small number of cases with minimum of 2-year follow-up duration. Third, sagittal plane assessment was not performed in this study. The implant shape was designed as mediolateral tapered and anteroposterior flat stem. Considering the less cortical contact in sagittal plane, stress shielding might not occur in such cross-section surface. Future studies with larger sample size and longer follow-up duration are required to validate long-term significance.

## 5. Conclusions

The manner of stem fitting defined as ML fit (forty-six or forty-two cases), FLR fit (nine or four cases), and MULTI fit (five or fourteen cases) showed different contact regions in 13 of 60 cases by using 2D or 3D analysis, respectively. This study successfully revealed that the ML fitting with femoral component, especially the most proximedial calcar site restricted fitting will be the best, would be optimal for reducing stress shielding occurrence in THA. Further research will be needed to confirm our findings presented in this study.

## Figures and Tables

**Table 1:** Associations between stress shielding degree and femoral stem contact sites.

	SS Degree 0 and 1 (*n* = 29)	SS Degree 2 (*n* = 13)
2 fitting areas, *n*	12 (41.4%)	2 (15.4%) ^NS^
3 fitting areas, *n*	15 (51.7%)	5 (38.5%) ^NS^
4 or more fitting areas, *n*	2 (6.9%)	6 (46.2%) **

** *p* < 0.01 vs. SS degree 0 and 1 cases (chi-squared test). SS, stress shielding; NS, not significant.

## Data Availability

The datasets generated during and/or analyzed during the current study are not publicly accessible but are available from the corresponding author on reasonable request.

## Supplementary Materials

The following supporting information can be downloaded at: https://www.mdpi.com/article/10.3390/jcm12093138/s1, Figure S1: Workflow of the present study.
