# Supplementary figures and images for: Relationship between Stress Shielding and Optimal Femoral Canal Contact Regions for Short, Tapered-Wedge Stem Analyzed by 2D and 3D Systems in Total Hip Arthroplasty
Source: J Clin Med. 2023 Apr 26;12(9):3138. doi: 10.3390/jcm12093138 (PMC10179674; doi:10.3390/jcm12093138)

## Supplemental Figure S1. Workflow of the present study.

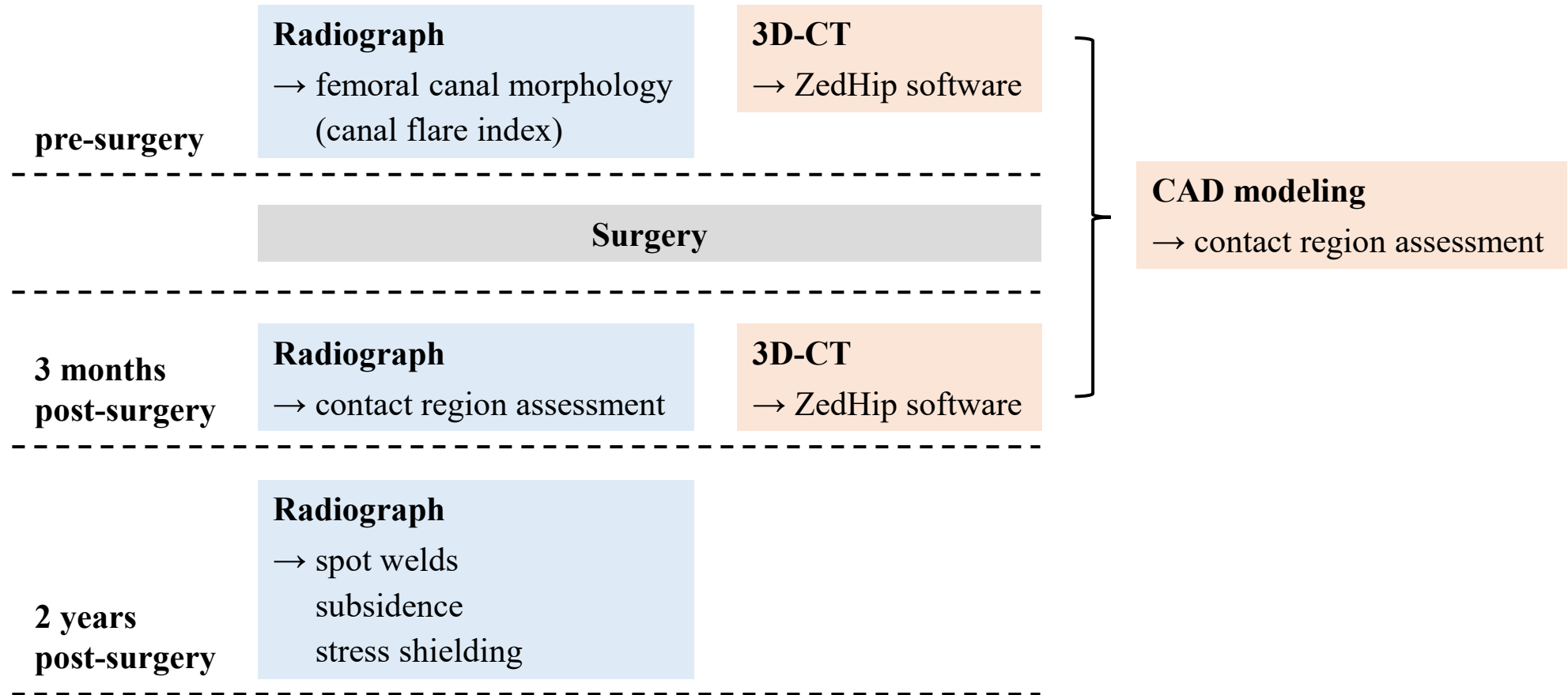

Supplement: Supplementary file 1 [file jcm-12-03138-s001.zip › jcm-2250260-supplementary.pdf]
